# An Actual Insight into the Pathogenic Pathways of Ankylosing Spondylitis

**DOI:** 10.3390/cimb46110762

**Published:** 2024-11-11

**Authors:** Emilia-Daniela Păsăran, Andreea Elena Diaconu, Corina Oancea, Andra-Rodica Bălănescu, Sorina Maria Aurelian, Corina Homentcovschi

**Affiliations:** 1 Faculty of Medicine, ‘Carol Davila’ University of Medicine and Pharmacy, 020021 Bucharest, Romania; emilia-daniela.pasaran@drd.umfcd.ro (E.-D.P.); andreea-elena.savulescu@drd.umfcd.ro (A.E.D.); sorina.aurelian@umfcd.ro (S.M.A.); corina.homentcovschi@umfcd.ro (C.H.); 2 Department of Physical Medicine and Rehabilitation, ‘Carol Davila’ University of Medicine and Pharmacy, 020021 Bucharest, Romania; 3 Department of Internal Medicine, ‘Carol Davila’ University of Medicine and Pharmacy, 020021 Bucharest, Romania; 4 Department of Medical Semiology, ‘Carol Davila’ University of Medicine and Pharmacy, 020021 Bucharest, Romania

**Keywords:** ankylosing spondylitis, pathogenesis, HLA-B27 antigen, genetics, immune system

## Abstract

Spondyloarthritis refers to a broad group of conditions that include ankylosing spondylitis, psoriatic arthritis, reactive arthritis, and enteropathic arthritis associated with Crohn’s disease or ulcerative colitis. They have been classified by the ASAS group (ASsessment in Ankylosing Spondylitis) into axial spondyloarthritis and peripheral spondyloarthritis. Common features include the absence of autoantibodies, genetic predisposition, and clinical aspects such as axial joint involvement, peripheral manifestations, and extra-articular involvement. However, the pathogenic mechanisms remain complex and incompletely elucidated, despite the fact that the specialized literature has described several pathways that act in synergy: genetic predisposition, environmental factors (infections and mechanical stress), or innate and acquired immune mechanisms. Finally, an inflammatory response is triggered by the recruitment of a large number of inflammatory cells and the release of innate cytokines in the affected areas: joints or periarticular or extraarticular tissues. The current article aims to update and systematize the knowledge accumulated so far on this topic, focusing on the mechanisms that have been involved in the onset, progression, and severity of ankylosing spondylitis.

## 1. Introduction

Spondyloarthritis refers to a broad group of conditions that include ankylosing spondylitis, psoriatic arthritis, reactive arthritis, and arthritis associated with inflammatory bowel diseases. Depending on the predominant manifestations and the need for early treatment, these have been classified by the ASAS (ASsessment in Ankylosing Spondylitis) group into axial spondyloarthritis (non-radiographic axial spondyloarthritis and ankylosing spondylitis) and peripheral spondyloarthritis (psoriatic arthropathy, reactive arthritis, spondyloarthritis associated with inflammatory bowel disease, and juvenile spondyloarthritis). Common features include the absence of autoantibodies, genetic predisposition, and clinical aspects such as axial joint involvement, peripheral manifestations, and extra-articular involvement [1,2].

The representative and most studied subtype of this class is ankylosing spondylitis (AS), which mainly affects the axial spine and sacroiliac and peripheral joints [3]. It is characterized by chronic back pain and reduced spinal mobility, changes such as erosions with bony growths typical of bilateral sacroiliitis, syndesmophytes, ankylosis of interapophyseal joints, calcifications of interspinal ligaments, progressive ankylosis, and disability [4,5]. It may be associated with peripheral joint manifestations predominantly seen in the lower limbs, such as arthritis, enthesitis, and dactylitis, all defining features of peripheral spondyloarthritis [6].

Over time, progress has been made in understanding the etiopathogenesis in order to provide patients with more targeted and effective treatment. Although still not fully understood, the etiopathogenesis of spondyloarthritis, and more specifically AS, has been described as involving several mechanisms of action that synergistically initiate and maintain a pro-inflammatory state, leading to disease onset. Genetic predisposition plays an essential role in conjunction with environmental factors, infections, and mechanical stress at the entheses, as well as the body’s immune response to triggering factors through innate and acquired immune mechanisms. Thus, several hypotheses have been put forward: the arthritogenic peptide hypothesis, the molecular mimicry hypothesis, the abnormal folding hypothesis of the human leucocyte antigen (HLA-B27) molecule in the endoplasmic reticulum, and the hypothesis of forming stable homodimers without involving the light chain, i.e., beta2-microglobulin [7]. 

## 2. Pathogenic Mechanisms

**Figure 1:**
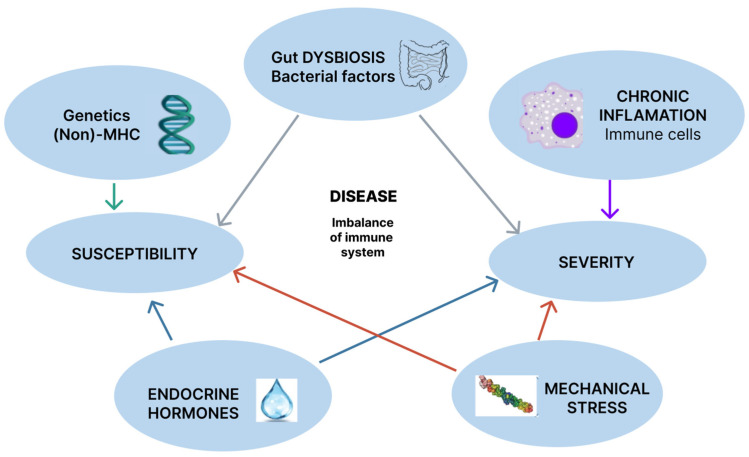
The key mechanisms for understanding the pathogenesis of ankylosing spondylitis.

The essential mechanisms in understanding SA pathogenesis are presented in Figure 1.

### 2.1. Genetics

#### 2.1.1. HLA-B27 (MHC Genetics)

Genetic predisposition refers to the primary role played by the HLA-B27 antigen in the onset of the disease. This theory was first described in 1950, but it was only in 1973 that the importance of its presence was observed in patients who developed the disease. In the general population, a positivity rate of 6–8% for the HLA-B27 antigen has been observed and is more prevalent at northern latitudes (which also highlights the genetic factor, given its presence in more than 80% of patients with AS [8,9,10,11]).

The human leukocyte antigen is a member of the class of alloantigens and is encoded by genes located in a region called the major histocompatibility complex (MHC), situated on the short arm of chromosome 6. The MHC region has three subgroups: class I, II, and III MHC. HLA-B27 antigen is associated with class I MHC, whose role is to present endogenous antigens to CD8+ T lymphocytes. It consists of two components: a heavy alpha chain and a light beta2-microglobulin chain. What differentiates it from other HLA-B molecules is that HLA-B27 contains a free cysteine at position 67 (cys67), through which disulfide bonds create stable homodimers without involving the light chain. On the other hand, the free light chain can be released by HLA-B27 and deposited in synovial tissue, stimulating inflammation in this area, or can interact with immunoreceptors—killer cell immunoglobulin-like receptors (KIR) and leukocyte immunoglobulin-like receptors (LILR)—expressed by inflammatory cells such as CD4+ T cells, NK cells, and myelomonocytes [10,12].

Another theory is that the abnormal organization of the HLA-B27 molecule, which is misfolded and accumulates in the endoplasmic reticulum, leads to an abnormal response. Misfolded proteins can be eliminated through autophagy or degradation. Three membrane proteins in the endoplasmic reticulum are involved: IRE1, PERK, and ARF6. Interaction with chaperones, which help incorrectly folded proteins achieve a proper tertiary structure activates the stress response. Inflammation is stimulated through specific receptors, the activation of the transcription factor NF-kB, as well as through the activation of the Interleukin (IL)-23/Interleukin (IL)-17 signaling pathway and the stimulation of cytokine production, including IL-17, tumor necrosis factor (TNF), and IL-6 [9].

HLA-B27 seems to have protective effects in HCV, HBV, and HIV infections, and some of the mechanisms (e.g., immune cell activation) responsible for AS seem to be involved in this ability to control the disease.

In HLA-B27-positive patients, significantly fewer HBV infections compared to in HLA-B27-negative patients were reported, suggesting that rheumatic patients have a better HBV control [13].

Research has highlighted the role of the immune system in controlling HIV infection. HLA-B27 and HLA-B57 are specific alleles associated with better infection control. The interaction of these genetic factors and host antiviral restriction mechanisms contribute to the varied clinical outcomes observed in HIV-1 patients [14].

HLA-B27’s intrinsic properties limit its therapeutic potential but HLA-B57:01:01 was identified as a promising candidate for a novel therapy, IOS-1002, designed in a format able to enhance stability and receptor binding. IOS-1002 binds to LILRB1, LILRB2, and KIR3DL1 receptors, potentially acting as a checkpoint blockade inhibitor. It demonstrates superior binding affinity, enhances macrophage phagocytosis, and down-modulates immunosuppressive signaling without requiring secondary activation signals. The molecule’s ability to simultaneously target multiple receptors may prevent tumor resistance [15].

#### 2.1.2. MHC Non-HLA-B27

Several MHC 1 or 2 loci associated with AS have been described, in addition to HLA-B27: HLA-B40, HLA-B60, HLA-A, HLA-DQA1, HLA-DRB1, and HLA-DPB1 [16]. HLA-A0201, identified with the single nucleotide polymorphism (SNP) *rs2394250*, was associated with AS, independently of the presence of HLA-B27. The mechanisms by which these MHC alleles cause AS remain unknown [17].

#### 2.1.3. Non-MHC Genetics

##### ERAP1/ERAP2

After the antigen was recognized, class I MHC molecules present the antigen to CD8+ lymphocytes. To be captured and transferred into the endoplasmic reticulum, the peptides must be cleaved to a length of approximately 15 amino acids [9].

This role belongs to endoplasmic reticulum aminopeptidases (ERAP) 1 and 2, and the genetic polymorphism of these two zinc metalloproteases represents the second major risk factor in the development of SpA, after the presence of HLA-B27 and it is considered to be responsible for approximately 7% of the risk of inheriting the disease. Together with HLA-B27, ERAP1 is accountable for 70% of genetic factors for familial AS. Considering its function, ERAP1 may contribute to abnormal peptide processing or antigen mispresentation, which leads to a predisposition to AS. To date, five single nucleotide polymorphisms (SNPs) of ERAP1 have been recognized: *rs27044*, *rs30187*, *rs2287987*, *rs10050860*, and *rs174820*. Other research has shown genetic interaction epistasis-like between ERAP1 SNPs, such as *rs30187*, *rs27044*, and *rs27037*, and HLA-B27.

ERAP1 is considered to be of great importance, as its role is to trim peptides stored in the endoplasmic reticulum, primarily at the N-terminal ends and hydrophobic residues at the C-terminal ends, adjusting their length so they can bind to MHC I and be presented on the surface of CD8+ T cells and NK cells. Additionally, ERAP1 cleaves cytokine receptors such as TNFR1, IL6R2, and IL1R2, thus modulating the availability of receptors on the cell surface. The genetic association of ERAP1 with AS was found in HLA-B27-positive individuals, suggesting that ERAP1 likely interacts with HLA-B27 and contributes to AS development through loss of function, aberrant processing, and defective antigen presentation. Furthermore, interactions with ERAP1 have been discovered in Behcet’s disease in association with HLA-B51, and in psoriasis in association with HLA-Cw6. The suppression of ERAP1/ERAP2 may represent a new therapeutic target in the future [11].

##### Other Non-MHC Genetic Mechanisms

Another class of genes that drew attention in the pathogenesis of AS includes GPR25, GPR35, and GPR65. In studies, it was observed that mice lacking GPR65, which then had Th17 cells activated by IL-23, result in a reduced production in IL-17A and an increased production in IL-10, indicating potential interest for future studies [18,19,20].

Another factor that should be mentioned in the pathogenesis of AS is genes modulating the activation and differentiation of either CD4 + or CD8 + T lymphocytes. Recent genome-wide association studies (GWASs) identified several types of genetic variation (SNPs, non-MHC genes that are connected with the production as well as activation of lymphocytes in AS, such as RUNX3, EOMES, ZMIZ1, IL7, TBX21, and IL7R. Runt-related transcription factor 3 (RUNX3), which belongs to the transcription factor family, can stimulate T cell differentiation to CD8+ T lymphocytes. RUNX3 polymorphisms have been linked to many human immune diseases or inflammatory processes, including systemic lupus erythematosus, psoriatic arthritis, and AS. Previous studies have revealed a connection between the RUNX3 polymorphism *rs11249215* and AS in Caucasian, Han Chinese, and Korean populations, and *rs4648889* has been related to reduced RUNX3 expression in AS. RUNX3 stimulates eomesodermin expression encoded by the EOMES gene and is the transcription factor related to the differentiation of CD8. In addition to RUNX3, research has suggested a relationship between these AS-associated non-MHC genes and lymphocyte differentiation or activation. Polymorphisms of molecules involved in the activation or suppression of lymphocytes, such as programmed cell death 1 (PDCD1), encoding PD-1, or T lymphocyte antigen 4 (CTLA-4), encoding CTLA-4, have been shown to influence the susceptibility to AS [21].

Autophagy is recognized as an important mechanism for physiological activity and cellular homeostasis characterized by cellular degradation of its own components [22]. The expression and significance of autophagy-related mRNA and long non-coding RNA (lncRNA) GAS5 in peripheral blood mononuclear cells (PBMCs) of patients with AS was recently investigated. The mRNA levels of several autophagy genes (LC3, Beclin1, ATG3, ATG5, ATG12, ATG16L1) and lncRNA GAS5 were measured using reverse transcription-quantitative PCR. Results showed that AS patients exhibited lower expression levels of these genes compared to healthy controls (HCs), particularly in active disease cases. Notably, ATG5 and ATG12 levels were negatively correlated with disease activity, while lncRNA GAS5 positively correlated with other autophagy gene expressions. The decreased expression of autophagy genes and the identification of lncRNA GAS5 may have potential implications for both diagnosis and disease severity assessment [22].

Ferroptosis is a regulated form of cell death, specifically driven by metabolic changes and characterized by the accumulation of lipid peroxides and iron-dependent oxidative stress [23].

During the inflammatory process, changes in serum levels of iron, anemia, thiols, protein oxidation, and lipid peroxidation were observed, suggesting their role in the etiopathogenesis of AS [24,25].

Iron accumulates at the intracellular, mitochondrial level, impairing mitochondrial function, but it also serves as a catalyst and cofactor for lipoxygenase, being involved in lipid peroxidation. Arachidonic acid, a polyunsaturated fatty acid, is the main substrate for this process, while lipid compounds (leukotrienes) represent the important pro-inflammatory metabolites produced, and reactive oxygen species may be generated as a secondary result. In patients with AS, high levels of intracellular iron were observed in PMNs, positively correlated with the level of inflammation and markers of oxidative stress in the serum of patients with AS and metabolic syndrome, as well as an increased level of reactive species of oxygen in the adjacent tissue to the vertebral body [26]. 

Two ferroptosis-associated genes (FRGs) were revealed, DDIT3 and HSPB1, and related to immune cell accumulation. 

Findings indicated that DDIT3 correlated positively with the infiltration of various immune cells, while HSPB1 showed a negative correlation. Both genes were recognized as diagnostic markers and potential therapeutic targets for AS, suggesting they may influence ferroptosis and modulate the inflammatory response in the immune microenvironment [27]. Further research is needed to elucidate the precise mechanisms and to explore ferroptosis as a potential therapeutic target in AS management.

Pyroptosis is a form of regulated cell death, characterized by inflammation and the release of pro-inflammatory cytokines. It is generally associated with the activation of inflammasomes, a cytosolic molecular platform, which activates the Caspase-1 family of proteases, resulting in the release of the cytokines IL-1β, IL-18, and gasdermin D, which act as the main executors of cell death. This can amplify the inflammation, contributing to the chronic nature of the disease [28,29,30].

Thus, an indirect link between AS and the NLRP3 inflammasome was observed, with significantly higher Caspase-1 levels in AS patients and elevated CRP levels compared to other types of inflammatory arthritis; however, other studies remain of interest. Elevated levels of NLRP3 and the ASC gene, along with NLRP3 and specific inflammatory cytokines, have been observed in AS patients, highlighting their potential as important therapeutic targets for the future [31].

The genetic factor has also been studied, and sixteen different pyroptosis genes have been identified in AS patients. These genes have been linked to immune cells (e.g., CD8+ T cells and neutrophils), IL-1β, and TNF signaling pathways.

AS patients were classified into two pyroptosis subtypes based on pyroptosis genes, with significant differences in immune infiltration. Molecular studies have revealed strong binding affinity between one of these genes, GZMB, and certain antioxidant drugs such as ascorbic acid or celastrol, providing arguments for potential future therapeutic targets [32].

These non-MHC gene variants function in the pathogenesis of AS via insufficiently elucidated mechanisms, and further investigations are needed regarding their pathways and possible clinical applications [21].

### 2.2. Environmental Factors

#### 2.2.1. Gut Dysbiosis

Composed of billions of bacteria, gut microbiota play an important role in digestion and defense against infections, thus maintaining the integrity of the intestinal barrier. The literature suggests that alterations in the intestinal flora are correlated with autoimmune diseases, molecular mimicry, and the activation of innate immunity through the recognition of non-self particles by antigen-presenting cells [33].

The pathogenic mechanisms remain complex and incompletely elucidated at present. The onset of the inflammatory response occurs following the changes made by the aggressive agent, as well as due to the recruitment of a large number of inflammatory cells to the affected site. Considering these, two theories have been supported: the recognition of exogenous molecules of infectious origin, called PAMPs (pathogen-associated molecular patterns), and the recognition of endogenous molecules released by damaged cells, called DAMPs (damage-associated molecular patterns) [34,35].

Antigen-presenting cells (APCs) recognize pathogens (bacteria or viruses) through specific receptors (PRR-pattern recognition receptors), capture and phagocytose antigens under the action of lysosomal enzymes, and release the epitope (which binds to the T cell receptor, TCR). Additionally, in APCs, the antigen associates with MHC in order to be recognized by T cells. In this process, lymphocyte receptors are also activated, acting as adhesion molecules (such as the intracellular adhesion molecule ICAM; the leukocyte function-associated antigen-3 (LFA-3) on the APC membrane; and CD2 and LFA-1 on the lymphocyte membrane), which ensure coupling between the APC and the T cell, activate the costimulatory signal, and release cytokines that facilitate communication between inflammatory cells. T cell activation leads to the clonal expansion and the perpetuation of inflammation. Through various pathways, HLA-B27 activates innate immunity and alters the permeability of the intestinal wall. Differences in the gut microbiota of HLA-B27-positive patients compared to HLA-B27-negative patients were observed, suggesting the importance of genetic factors [11].

Diverse studies have supported the idea of an immunomodulatory role of certain bacterial agents. In the gut microbiota, an increase in the *Ruminococcus gnavus*, *Clostridium symbiosum*, *Clostridium bolteae*, and *Erysipelatoclostridium ramosum* species were observed in patients with severe AS compared to those with mild disease, who exhibited a predominance of *Firmicutes* species. Additionally, *Ruminococcus gnavus* was also present in patients with IBD, suggesting a possible common factor between the two conditions [35].

The theory of molecular mimicry and arthritogenic peptide also apply at this level. There are certain microbial peptides that are structurally similar to the body’s own peptides and are recognized by CD8+ T cells through HLA-B27, creating a phenomenon of cross-reactivity. Examples include: *Klebsiella pneumoniae*, *Yersinia enterocolitica* and *pseudotuberculosis*, *Shigella flexneri*, *Salmonella typhimurium*, and members of the *Enterobacteriaceae* family [36]. Additionally, *Salmonella* occupies altered peripheral cells and activates inflammation in the intestine in the presence of abnormal folding of HLA-B27:05 in the endoplasmic reticulum. This mechanism was observed in reactive arthritis [37]. Increased levels of *Ruminococcus gnavus* and changes in the commensal flora have been observed in patients with SpA and Crohn’s disease, which are less pronounced in patients with RA [38].

In the case of infection with *Candida albicans*, it is presumed that inflammation is stimulated through the secretion of IL-17, increased intestinal permeability, and molecular mimicry in certain species, leading to the onset of spondyloarthritis, inflammatory bowel disease (IBD), or psoriasis. The risk of developing AS appears after a duration of 6 years [39]. 

The hypothesis has also been supported by the fact that the bacterial agent migrates through the lymphatic system, connecting the regional lymph nodes located in the lower intestinal tract, those in the pelvic floor, and the sacroiliac joints. It has been observed that cells expressing specific intestinal tract markers are also found in the joints [40]. IL-23, produced in the small intestine and activated at the mucosal level, plays an important role in activating the innate immune system by activating a group of lymphoid cells called type 3 innate lymphoid cells (ILC3). Originating from the intestinal mucosa, the α4β7+ ILC3 subtype extends into the bloodstream and to the joints and is responsible for producing IL-17 and IL-22 [41,42].

The involvement of multiple pathogens and different mechanisms suggests the complexity of the gut microbiota and its importance in the etiopathogenesis of spondyloarthritis.

#### 2.2.2. Mechanical Stress

Mechanical stress is an important factor in triggering SpA, which occurs at the entheses, the insertion points of tendons, ligaments, and the capsule into the bone. Due to mechanical overload, the entheses of the lower limbs are more frequently affected compared to those of the upper limbs. Mechanical overuse leads to the production of microtraumas, releasing arthritogenic peptides derived from cartilage, such as fibronectin and hyaluronate, which increases local vascularization, an influx of inflammatory cells, erosions, and new bone formation. The question has been raised as to why inflammation in spondyloarthritis begins at the entheses. It was observed in animal models that there is dysfunction either in the TNF-α pathway or the IL-23/IL-17 pathway.

At the entheses, increased levels of resident lymphocytes of the CD3+CD4-CD8- type expressing IL-23R have been observed. More recently discovered in animal models were γδ T cells, particularly the Vδ1 T cells and Vδ2 T cells subtypes, which can produce IL-17A under the action of IL-23 [43,44]. Cuthbert et al. demonstrate the presence of these cell populations both in normal human entheses and in affected entheses. The Vδ2 T cells population shows increased levels of transcription factors such as RORC, IL-23R, and chemokine receptor CCR6, which are involved in IL-17/IL-23 axis signaling. The study confirms that the Vδ1 subtype does not express IL-23R in enthesis tissue under normal conditions, and the production of IL-17A, IL-17F, and IL-22 can be induced in the absence of IL-23. However, it cannot be excluded as a determining factor under pathological disease conditions [45].

On the other hand, type II collagen is the primary component of articular cartilage. Its degradation by collagenases triggers chondrocytes to synthesize procollagen, resulting in an increase in the C-propeptide of type II collagen (CPII). Kim TH et al. utilized two markers for cartilage matrix formation and turnover (the 846 epitopes of aggrecan and CPII), as well as two markers indicating joint degradation (C2C and C1-2C, which are epitopes from type II collagen degradation). They also measured various inflammatory interleukins in patients with AS undergoing treatment with Infliximab to assess disease activity and response to treatment. Elevated levels of CPII and the 846 epitopes were found in AS patients. The correlation of the CPII ratio with CRP values was informative, but it requires further study and remains of interest [46].

### 2.3. Chronic Inflammation

#### 2.3.1. Immune Cells

Under stressful conditions, such as mechanical stress, gut microbiome changes, or environmental factors, AS is linked to chronic inflammation that involves dendritic cells, macrophages, NK cells, and adaptive immune cells. These immune cells release a range of innate cytokines and play a role in the initiation and progression of the disease [3].

Dendritic cells (DCs) are crucial in the immune response as they initiate and guide the inflammatory process. Derived from lymphoid and non-lymphoid organs, these cells are of two types: the CD1c+ DC1 subset and the CD141+ DC2 subset. CD1c+ induces Th1 and Th2 responses. The decrease in the number of DCs was accompanied by an increase in a mononuclear cell line, especially CD14-CD16+ monocytes, leading to the secretion of IL-1β and IL-6 and the stimulation of Th17 cells. Changes have also been observed in the function and the gene expression of these cells in patients with AS who are HLA-B27-positive [47].

Macrophage cells (Mf), which function as phagocytes and antigen-presenting cells, represent a cell lineage involved in cellular defense. Two types of macrophages were identified: M1 effector macrophages, which play a role in cellular defense and secrete various interleukins, including IL-1β, IL-6, IL-12, IL-23, and TNF, and M2 regulatory macrophages, which help to reduce the inflammatory response. In patients with spondyloarthritis (SpA), a predominance of the M2 subtype over M1 was observed at the synovial level, which distinguishes the synovium of patients with SpA from that of patients with rheumatoid arthritis (RA), which has a higher frequency of Th17 cells. These macrophages show the increased expression of HLA-DR and secrete TNF, thus perpetuating inflammation [48,49]. The number of macrophages in patients with AS is closely related to the severity of the disease. Previous studies have confirmed its importance in inflamed tissues in patients with SpA and in particular the main role of CD163 + and CD68 + macrophages, as well as activated osteoclasts, which have also been observed in the sacroiliac joints [50].

Natural killer (NK) cells, a subset of lymphocytes, play a role in defense against viruses, bacteria, and cancer cells, without requiring prior activation. Although less prevalent in autoimmune diseases, they are found in large numbers in inflammatory diseases. In addition to their surface markers, most of which are CD56+, NK cells contain a large number of cytoplasmic granules that hold their lytic arsenal, including perforins and granzymes. The recognition of target cells is mediated through the NK cell receptor (killer-cell immunoglobulin-like receptor, KIR), which has two components: an activating receptor that binds to a ligand on the surface of nucleated cells in the body, and an inhibitory receptor that binds to MHC I molecules. Considering the importance of HLA-B27 in AS, among the KIR genes, KIR3DL1/3DS1 has drawn attention due to its recognition of HLA-B27, although further larger studies are needed in AS patients [47,51,52].

#### 2.3.2. The IL-23/IL-17 Axis

The dysfunction of the IL-23/IL-17 pathway has been observed in various autoimmune disorders, including psoriasis, rheumatoid arthritis, inflammatory bowel disease (IBD), and spondylarthritis (SpA). Additionally, blocking the IL-23/IL-17 pathway has been shown to significantly improve AS. In AS, differentiated T lymphocytes produce IL-17, which activates osteoclasts and inhibits bone regeneration. Meanwhile, these lymphocytes can also release IL-22 in response to IL-23, promoting osteoproliferation. This opposing process may account for the simultaneous occurrence of bone erosion and formation in patients with AS [53].

The cytokines IL-23 and IL-12, similar in structure but different in action, are part of the same IL-12 family of cytokines. Highly inflammatory, IL-23 is secreted by monocytes, macrophages (MFs) and dendritic cells (DCs) of peripheral tissues, including skin, joints, intestinal mucosa, and lungs. Unlike IL-12, which induces the differentiation of Th0 cells into Th1 cells through the production of Interferon (IFN)-γ, IL-23 promotes and maintains, thanks to a synergistic action with IL-1β and transforming growth factor (TGF)-β, the differentiation of CD4+ αβ T lymphocytes (Th0) into Th17 cells, leading to the production of IL-17, IL-6, IL-22, and TNF-α [54].

The alteration of the balance between Th1 and Th2 cell populations has been observed in patients with mild and severe forms of AS. Cytokines produced by Th1 cells, such as TNF-α and IFN-γ, perpetuate inflammation and are key molecules in the development of rheumatic diseases, while cytokines produced by Th2 cells, such as IL-4 and IL-10, attempt to suppress inflammation. The role of chemokines in the recruitment of cells to the wound site is also important. CXCL10, induced by IFN-γ, has been considered an aggressive marker of Th1-mediated inflammation, with high serum levels observed in patients with AS, perpetuating the production of IFN-γ and TNF-α [55,56] while CCL17 and CCL22 try to restore the balance between Th1 and Th2 cells and play a role in the Th2 type response. An increased Th2-like response was also associated with the overexpression of the chemokine receptor CCR4 on CD4+ T cells, which was positively correlated with BASDAI (Bath Ankylosing Spondylitis Disease Activity Index) in AS patients [47].

IL-17 is a cytokine family that includes six members, from IL-17A to IL-17F, with IL-17A being the main product of Th17 cells. Under normal conditions, this molecule plays a role in antimicrobial defense by initiating chemotaxis, recruiting and activating neutrophils to the wound site, and inducing the production of angiogenic factors and matrix metalloproteinases. In the inflammatory process, in addition to TNF-α, it increases the pro-inflammatory response by increasing the production of IL-6, TNF-α, and IL-1β. Regarding bone metabolism, IL-17 has a bone resorption effect through the activation of NF-κB ligand (RANKL), the stimulation of osteoclasts and osteoclastogenesis, and induces macrophages to produce matrix metalloproteinases (MMPs) [54].

Several additional loci associated with the IL-23/IL-17 pathway have been identified, including IL-1R1, IL-2R, IL-6R, IL-12B, IL-27, STAT3, RUNX3, CARD9, and TYK2 [12]. Given these findings, it makes sense to focus further on the relationship between IL-23/IL-17 stimulation and AS, as well as potential therapeutic targets along the IL-23/IL-17 axis [57].

### 2.4. Endocrine Hormones

#### 2.4.1. Vitamin D

In immune-mediated rheumatic diseases, vitamin D plays a protective role, and vitamin D deficiency has been considered a risk factor associated with more severe disease [58]. Vitamin D is a hormone involved in osteoblast activity and plays a role in pathogenic mechanisms by regulating the balance of Th1/Th2 cells in favor of Th2 cells [59]. It also participates in the regulation of TNF-α production by macrophages [60]. In patients with AS, BASDAI score and inflammatory markers were strongly associated with vitamin D levels. Higher vitamin D levels associated with lower disease activity support its protective role [59].

#### 2.4.2. Sex Hormones

The role of the endocrine system in the functioning of both innate and adaptive immunity is recognized, but evidence linking these two domains is scarce [61,62]. Sex hormones are also involved in the pathogenesis of AS. The disease is more common in men and more active and more common in women after childbirth. Low levels of estradiol have been observed in women with active disease, and there may be a possible link between low levels of dehydroepiandrosterone sulfate (DHEA-S) and decreased bone density in men [21,63]. Estrogens are involved in T cell differentiation and even in some cytokine production—for Type 2 cytokine, some data also point to the effect of downshifting the T helper 17 differentiation. In an animal model, estrogens decrease arthritis development, probably through a complex cellular communication pathway, Wnt signaling [64].

Table 1 summarizes the pathogenic mechanisms involved in the occurrence, progression, and severity of ankylosing spondylitis.

## 3. Conclusions

Variations in HLA-B27 and ERAP 1 are genetically associated with AS susceptibility. The inflammatory process defined by the activation, the accumulation of immune cells (dendritic cells, macrophages, and natural killer cells) with the secretion of cytokines is the main component involved in the progression and the severity of the disease. Most known dates are related to a high Th1 density, an increased Th1/Th2 fraction and the IL-23/IL-17 pathway. Elevated serum levels of IL-23 and IL-17 as well as IL-23R genetic polymorphism were found in AS patients. 

Recent data mention the role of the endocrine system, referring to the influence of sex hormones or vitamin D. In favor of the possible role of sex hormones in the occurrence of AS are male predominance and some results of observational studies which showed an increased number of first manifestations after pregnancy. Meta-analyses have suggested that vitamin D deficiency may be associated with the development of AS; therefore, vitamin D may play a protective role in AS.

Knowing the pathophysiological mechanism that contributes to the onset and progression of AS is important for understanding the varied responses to therapies among individuals and is crucial in searching new solutions. As it can be seen, this increasingly large puzzle of information complicates the direction of research, highlighting the need to focus on core essentials to facilitate progress in the field. From this perspective, it is vital to explore different therapeutic areas that combine immunological treatments with other types of interventions.

There are numerous emerging areas of interest in research, including the development of new monoclonal antibodies and environmental approaches, such as dietary changes and supplements aimed at improving the microbiome, which require specific studies to be conducted promptly. There is also an urgent need for genetic solutions and studies focused on optimizing treatment for individual patients, potentially aided by AI. Personalized combination therapies represent the future of effective management for AS patients.

## Figures and Tables

**Table 1:** Pathogenic mechanisms involved in the occurrence, progression, and severity of ankylosing spondylitis.

Type of Mechanism	Marker	Basis	Pathway	Role
**Genetics**	HLA-B27 and other MHC alleles	Polymorphism	Peptide binding specificity, misfolding, and forming heavy chain homodimers;	Susceptibility
	Non-MHC—ERAP1	Polymorphism	Alterations in peptide and cytokine receptors processing	Susceptibility
**Gut dysbiosis**	Ruminococcus gnavus and species of Clostridiales (Erysipelatoclostridium ramosum, Clostridium symbiosum, Clostridium bolteae)	PAMPs, DAMPs	Changes in the gut microbiome, inappropriate immune responses	Susceptibility and severity
**Mechanical stress**	CD3+CD4-CD8-type expressing IL-23R lymphocytes, Epitopes from type II collagen degradation	Microtraumas releasing arthritogenic peptides	Dysfunction in the TNF-α or IL-23/IL-17 pathway	Trigger and disease activity
**Chronic inflammation**	High Th1 density, Increased Th1/Th2 fraction, IL-23/IL-17 pathway, IL-23R genetic polymorphism	Changes in cell response	Activation and accumulation of immune cells (dendritic cells, macrophages, and natural killer cells) with the secretion of cytokines	Progression and severity
**Endocrine hormones**	Sex hormones, Vitamin D, Modulators of the Wnt pathway	Effects on the immune system	Regulating the balance of Th1/Th2 and TNF-α production	Onset, disease activity, and severity

## Abbreviations

APCs—antigen-presenting cells; ARF6—ADP-Ribosylation Factor 6; AS—ankylosing spondylitis; ASAS—ASsessment in Ankylosing Spondylitis; ASC—apoptosis-associated speck-like protein containing a CARD; ATGs—autophagy-related genes; C1-2C—Collagen type I and II C-telopeptide; C2C—Collagen type II C-telopeptide; CARD9—Caspase Recruitment Domain 9; CCR6—C-C Chemokine Receptor Type 6; CD2—Cluster of Differentiation 2; CPII—C-propeptide of type II collagen; CTLA-4—cytotoxic T-lymphocyte-associated protein 4; DAMPs—damage-associated molecular patterns; DDIT3—DNA damage-inducible transcript 3; EOMES—eomesodermin; ERAP—endoplasmic reticulum aminopeptidases; FRGs—ferroptosis-associated genes; GAS5—growth arrest-specific 5; GZMB—granzyme B; HBV—hepatitis B virus; HCV—hepatitis C virus; HIV—human immunodeficiency virus; HLA—human leucocyte antigen; HSPB1—heat shock protein family B member 1; ICAM—intracellular adhesion molecule; IL—interleukin; ILC3—type 3 innate lymphoid cells; ILR—interleukin receptor; IRE1—inositol-requiring enzyme 1; KIR—killer cell immunoglobulin-like receptors; KIR3DL1—killer immunoglobulin-like receptor 3DL1; LC3—microtubule-associated protein 1A/1B-light chain 3; LFA-1—lymphocyte function-associated antigen 1; LFA-3—leukocyte function-associated antigen-3; LILR—leukocyte immunoglobulin-like receptors; LILRB1—leukocyte immunoglobulin-like receptor B1; LILRB2—leukocyte immunoglobulin-like receptor B2; MHC—major histocompatibility complex; NF-kB—nuclear factor kappa B; NLRP3—NOD-like receptor family pyrin domain containing 3; PAMPs—pathogen-associated molecular patterns; PDCD1—programmed cell death 1; PERK—protein kinase RNA-like endoplasmic reticulum kinase; PRR—pattern recognition receptors; RORC—retinoic acid receptor-related orphan receptor C; RUNX3—runt-related transcription factor 3; SNP—single nucleotide polymorphism; STAT3—signal transducer and activator of transcription 3; TBX21—T-box transcription factor 21; TCR—T cell receptor; TNF—tumor necrosis factor; TYK2—tyrosine kinase 2; Vδ1 T cells—subtype of T cells expressing the Vδ1 T cell receptor (TCR) chain; Vδ2 T cells—subtype of T cells expressing the Vδ2 T cell receptor (TCR) chain; ZMIZ1—zinc finger MIZ-type containing 1.
